# Factors Determining a Recommendation for Termination of Pregnancy in the Presence of Fetal Renal Anomalies

**DOI:** 10.3390/jcm15218620

**Published:** 2026-11-05

**Authors:** Abdulmecit Oktem, İlayda Gercik Arzik, Mukremin Ceylan, Zübeyde Emiralioglu Cakir, Orhan Nural, Atalay Ekin

**Affiliations:** 1 Department of Perinatology, University of Health Sciences Izmir City Hospital, Sevket Ince Neighborhood, 2148/11, 35540 Izmir, Turkey; ilgercik@gmail.com (İ.G.A.); mukreminceylan68@gmail.com (M.C.); drorhannural@gmail.com (O.N.); atalayekin@hotmail.com (A.E.); 2 Division of Perinatology, Department of Obstetrics and Gynecology, Buca Seyfi Demirsoy Education and Research Hospital, 35390 Izmir, Turkey; zubeydeemiralioglu@hotmail.com

**Keywords:** congenital anomalies of the kidney and urinary tract, fetal renal anomaly, prenatal counseling, termination of pregnancy, multidisciplinary fetal council, genetic testing

## Abstract

**Background/Objectives:** To identify the clinical factors associated with a multidisciplinary fetal council’s recommendation for termination of pregnancy in fetuses with renal anomalies, to describe how often families followed this recommendation, and to describe outcomes among continued pregnancies. **Methods:** This retrospective cohort study included 103 singleton pregnancies with a prenatally diagnosed renal or urinary tract anomaly discussed at a tertiary fetal council between December 2023 and July 2026. Renal severity, gestational age at diagnosis, amniotic fluid status, associated fetal abnormalities, and genetic evaluation were examined in relation to the council recommendation. Independent associations were assessed using multivariable logistic regression, Poisson regression with robust variance estimation, and Firth penalized logistic regression. Postnatal outcomes were summarized for pregnancies that continued. **Results:** A termination recommendation was issued in 59 of 103 pregnancies (57.3%). Of these, 43 families (72.9%) accepted it and the pregnancy was terminated, whereas 16 (27.1%) continued the pregnancy. Later diagnosis and moderate or low renal severity were independently associated with a lower likelihood of a termination recommendation. The presence of an additional fetal anomaly (adjusted odds ratio [aOR] 8.24, 95% confidence interval [CI] 1.59–42.65) and completion of genetic testing (aOR 4.53, 95% CI 1.45–14.15) were independently associated with a higher likelihood of such a recommendation. Among 60 continued pregnancies, 11 intrauterine or neonatal/postnatal deaths occurred (18.3%); mortality was 42.9% in the high-severity group and 5.3% in the low-severity group. The prenatal renal finding was confirmed postnatally in 22 cases, while renal-specific follow-up was unavailable in 18. **Conclusions:** Termination recommendations issued after prenatal detection of a renal anomaly were shaped by the timing of diagnosis, expected renal prognosis, the wider fetal phenotype, and the availability of genetic information. The observed difference between the council recommendation and the parental decision, together with substantial variation in postnatal outcomes, supports individualized counseling within a multidisciplinary framework.

## 1. Introduction

Congenital anomalies of the kidney and urinary tract encompass a heterogeneous group of abnormalities involving the kidneys, collecting systems, bladder, and urethra. They are among the most frequently identified fetal abnormalities on prenatal ultrasonography and account for a substantial proportion of childhood chronic kidney disease [1,2]. Their clinical course is equally broad: isolated urinary tract dilatation or a positional renal anomaly may have little long-term functional consequence, whereas bilateral renal agenesis, severe cystic disease, or lower urinary tract obstruction may lead to anhydramnios, pulmonary hypoplasia, kidney failure, or perinatal death [1,2,3].

Prenatal prognosis cannot be inferred from the diagnostic label alone. Laterality, the amount of functioning renal tissue, urinary obstruction, and amniotic fluid volume all modify the expected outcome, while additional structural abnormalities may indicate a syndromic condition and further worsen prognosis [3,4,5]. The genetic contribution is also increasingly recognized. Pathogenic copy-number variants and monogenic disorders are found in a meaningful proportion of fetuses with congenital kidney and urinary tract anomalies, with higher diagnostic yields in selected phenotypes and in non-isolated cases [2,6,7]. Genetic results can therefore refine recurrence counseling and postnatal planning, but they may also influence decisions made during an ongoing pregnancy.

Even after a detailed prenatal assessment, uncertainty often remains. Prenatal and postnatal diagnoses are not always concordant, particularly in milder or evolving urinary tract findings, whereas severe bilateral disease may be associated with early death or considerable neonatal morbidity [4,5,8]. Counseling consequently requires input from maternal–fetal medicine, pediatric nephrology, and medical genetics, with participation from other specialties when the renal finding is part of a broader fetal phenotype. A multidisciplinary setting allows families to receive a more integrated explanation of diagnostic uncertainty, expected survival, future kidney function, and the possible need for neonatal intervention [1,9].

Despite these advances, prenatal detection of renal anomalies remains imperfect. Visualization of the kidneys and bladder depends on gestational age, fetal position, amniotic fluid volume, and operator experience, so some lesions are recognized late and others are over- or under-called [1,4,5]. Prognosis is also uncertain, and no widely accepted guideline defines which prenatal features should lead to a recommendation to continue or to end a pregnancy, so advice may vary between centers and clinicians [1,3,9].

Most published cohorts have described diagnostic accuracy, genetic yield, pregnancy outcome, or postnatal renal prognosis. Less is known about the clinical considerations that shape the recommendation issued by a multidisciplinary fetal council before the family makes its final decision. We therefore evaluated pregnancies with prenatally diagnosed renal anomalies discussed at a tertiary fetal council. The primary aim was to identify factors independently associated with a recommendation for termination of pregnancy. We also examined how often families followed the recommendation, performed a separate analysis of isolated renal anomalies, and described postnatal outcomes according to prenatal renal severity.

## 2. Materials and Methods

### 2.1. Study Design and Population

We performed a retrospective review of the fetal council database at İzmir City Hospital, a tertiary referral hospital in Turkey. The source population comprised pregnancies referred to the council because prenatal ultrasonography had demonstrated a renal or urinary tract abnormality between December 2023 and July 2026. Eligible records were included consecutively. İzmir City Hospital began operating its multidisciplinary perinatology council only in late 2023; the study interval therefore spans the entire period for which council records exist at this institution, and no earlier data were available for inclusion.

The multidisciplinary fetal council routinely included specialists in maternal–fetal medicine, pediatric nephrology, and medical genetics. When additional fetal anomalies were present, specialists from the relevant clinical departments also participated according to the organ systems involved. For each referral, the council considered the complete prenatal phenotype rather than the renal diagnosis in isolation. The discussion incorporated the sonographic appearance of the kidneys and urinary tract, amniotic fluid volume, additional fetal findings, genetic information when available, and the likely consequences for survival and postnatal renal function. The recommendation recorded at the end of the meeting was coded as either continuation of pregnancy or termination of pregnancy. The recommendation constituted the main study outcome. Among families advised to terminate, acceptance or refusal of that advice was examined separately. Throughout this article, “termination recommendation” refers to the advice issued by the council, whereas “pregnancy termination” refers to the procedure actually carried out after the family’s decision. No formal published guideline specifies fixed criteria for recommending termination versus continuation in fetal renal anomalies; the recommendation instead reflected the consensus reached among all attending specialties after weighing the diagnosis, its laterality and severity, amniotic fluid status, associated findings, and overall prognosis for each individual case. Pregnancy termination in Turkey is regulated by the Population Planning Law (Law No. 2827); the legal framework is described in the Discussion.

Council records were screened consecutively. Inclusion required a sonographically identified renal or urinary tract abnormality, a documented council decision, and adequate information to determine both the principal diagnosis and its prenatal severity. Multiple pregnancies and records lacking the information necessary for classification were omitted. The sample therefore represented the complete series of eligible singleton pregnancies discussed during the study interval, and an a priori power calculation was not undertaken.

### 2.2. Data Collection and Definitions

Information was abstracted retrospectively from the hospital electronic record and the standardized council form. The study dataset contained maternal age, gravidity, parity, gestational age when the renal abnormality was diagnosed, the dominant renal diagnosis, amniotic fluid category, accompanying fetal findings, genetic testing status and result, the council decision, and the subsequent parental choice. Diagnostic gestational age was retained as a continuous measure and was also grouped as <20 weeks, 20–23 + 6 weeks, or ≥24 weeks to reflect clinically distinct decision periods. Parental sociodemographic characteristics, including educational attainment and household income, are not part of the standard hospital record and could not be analyzed. For each case, the council decision was determined by reviewing the written council minutes together with the corresponding entry in the electronic medical record.

The amniotic fluid assessment documented at diagnosis was recoded into four groups: anhydramnios, oligohydramnios, normal fluid volume, and polyhydramnios. Non-renal findings were assigned to the predominant clinical category: central nervous system, cardiac, gastrointestinal, skeletal or limb, hydrops/cystic hygroma, multiple or syndromic malformations, soft marker only, or none. For adjusted analyses, these findings were collapsed into a binary variable indicating the presence or absence of an additional anomaly. Cases without a non-renal structural abnormality were considered isolated renal anomalies.

Genetic testing was first coded according to whether an investigation had actually been completed; a test that was offered but declined was included in the untested group. Results were subsequently described as normal, pathogenic/abnormal, pending or unavailable, or not tested. All prenatal diagnostic procedures formed part of routine clinical care and followed genetic counseling and parental consent. Karyotyping and chromosomal microarray analysis, when performed, were carried out at the institution’s own cytogenetics and molecular genetics laboratory following standard clinical protocols. Whole-exome sequencing is not reimbursed by the national health insurance system in Turkey and, when pursued, was arranged privately by the family through an external commercial laboratory after separate informed consent. Variant results were classified according to the American College of Medical Genetics and Genomics (ACMG) criteria (pathogenic, likely pathogenic, variant of uncertain significance, likely benign, or benign).

### 2.3. Renal Anomaly Diagnosis and Prenatal Severity Classification

When more than one urinary finding was present, the lesion considered most relevant to prognosis was selected as the index diagnosis. The resulting diagnostic groups were lower urinary tract obstruction (posterior urethral valves or megacystis), horseshoe kidney, ectopic kidney, multicystic dysplastic kidney, polycystic kidney disease, renal agenesis, renal mass, ureteropelvic junction obstruction, isolated pyelectasis/hydronephrosis, and other renal abnormalities.

A three-level clinical severity variable was constructed from the prenatal assessment. Grading took account of bilateral versus unilateral involvement, the amount of functioning renal tissue, urinary obstruction, amniotic fluid volume, expected renal function, the probable need for neonatal treatment, and anticipated survival. The high-severity category denoted a risk of profound renal dysfunction, pulmonary hypoplasia, or death; the moderate category denoted a meaningful likelihood of postnatal morbidity, surveillance, or intervention; and the low category denoted an expected favorable course with limited functional impairment. As the prognosis of the same diagnostic entity may differ according to laterality and fluid status, severity was allocated individually rather than by applying a fixed grade to each diagnostic name. This grading drew on the prognostic principles underlying the Urinary Tract Dilation (UTD) consensus classification [10], extending them beyond isolated hydronephrosis to encompass the full spectrum of renal and urinary tract diagnoses evaluated by the council. The council applied no numerical cut-offs; severity was assigned by consensus for each case. The domains considered, with the characteristics and renal diagnoses observed in each category, are summarized in Appendix A (Table A1).

### 2.4. Follow-Up and Postnatal Outcomes

Delivery and neonatal data were sought for all ongoing pregnancies. The variables retrieved were gestational age at birth, Apgar scores at 1 and 5 min, birth weight, neonatal intensive care admission, fetal or neonatal/postnatal death, and whether the prenatal renal finding was verified after birth. Verification required documentation from postnatal imaging or clinical follow-up. If the postnatal assessment did not reproduce the prenatal finding, the result was classified as normal/not confirmed. Cases without sufficient renal follow-up were labeled as unavailable even when delivery data were present. Because neonatal intensive care status was missing for some infants, its frequency was calculated from the number with known information.

### 2.5. Statistical Analysis

Statistical analyses were performed using IBM SPSS Statistics version 26.0 (IBM Corp., Armonk, NY, USA). Firth penalized logistic regression (implemented in NumPy and SciPy), variance inflation factors, and Mantel–Haenszel estimates were computed in Python 3.13 (Python Software Foundation, Wilmington, DE, USA, statsmodels 0.15.0). Numerical variables are summarized by the median and interquartile range (IQR), and categorical variables by counts and percentages. Maternal and fetal characteristics were compared across the two council-decision groups. The Mann–Whitney U test was used for numerical variables, while categorical distributions were assessed with the chi-square test.

Independent associations with a recommendation for termination were estimated in a multivariable binary logistic model. The explanatory variables were diagnostic gestational-age group, renal severity level, any associated anomaly, and completion of genetic testing. These covariates were selected because of their clinical relevance and their relationship with the outcome in the initial comparisons. Effect estimates are given as adjusted odds ratios with 95% confidence intervals, and McFadden’s pseudo-R^2^ was used to describe model performance. All four candidate variables also showed a significant univariable association with the outcome (gestational-age category and completion of genetic testing, each *p* < 0.001; presence of an associated anomaly, *p* = 0.002; renal severity level, *p* = 0.020); maternal demographic and obstetric characteristics (age, gravidity, parity) were compared descriptively between groups but were not considered candidate explanatory variables for the model, as they are not established clinical determinants of fetal renal prognosis or of the council’s recommendation. As a sensitivity analysis, a second model included all variables with a univariable *p*-value below 0.10 (maternal age, parity, amniotic fluid status, and the four primary variables; gravidity was omitted because of its correlation with parity). Amniotic fluid status was not entered in the primary model because fluid volume is a component of the severity grade; the severity–fluid association was quantified with Cramér’s V and collinearity with variance inflation factors.

The outcome was common in this cohort; therefore, the same covariate set was also examined with a Poisson model using a sandwich variance estimator, allowing adjusted relative risks and 95% confidence intervals to be reported alongside odds ratios. A separate analysis was performed in the isolated-anomaly subgroup. For pregnancies continuing beyond the council decision, postnatal findings were described across the three prenatal severity levels. Available-case denominators were used where follow-up was incomplete. Statistical significance was defined by a two-sided *p*-value below 0.05. Because of the limited number of events and sparse cells, the models were also fitted by Firth penalized logistic regression. Confounding by indication for genetic testing was explored with a Mantel–Haenszel odds ratio stratified by additional anomaly, and postnatal outcomes were compared between recommendation groups with Fisher’s exact test.

### 2.6. Ethical Approval

The study protocol was approved by local non-interventional clinical research ethics committee (Approval no: 2026/34, Approval Date: 17 August 2026). The committee waived individual consent for this retrospective record review. Identifiers were removed from the analytic dataset, and all study procedures conformed to the Declaration of Helsinki.

During the revision, the authors used Claude Sonnet 5.5 (Anthropic San Francisco, CA, USA) for language editing and to support additional statistical analyses; all outputs were reviewed and verified by the authors.

## 3. Results

A total of 103 singleton pregnancies with a fetal renal anomaly were included (Figure 1). The fetal council recommended continuation of pregnancy in 44 cases (42.7%) and termination in 59 (57.3%).

Maternal and obstetric characteristics are summarized in Table 1. Maternal age did not differ significantly between the two recommendation groups (*p* = 0.060). Gravidity also differed between the groups (*p* = 0.017), but the median was 2 in both, so the difference lay in the spread of the distribution (interquartile range 2–3 vs. 1–3). Parity was lower among pregnancies for which termination was recommended (median 0 vs. 1, *p* = 0.003). Renal anomalies were diagnosed earlier in the termination-recommended group: the median gestational age at diagnosis was 21 weeks, compared with 25 weeks in the continuation-recommended group (*p* < 0.001). The contrast was also evident after categorization. Cases diagnosed before 20 weeks accounted for 39.0% of the termination-recommended group, whereas only 23.7% of this group had been diagnosed at or beyond 24 weeks (*p* < 0.001). Among the 59 families advised to terminate the pregnancy, 43 (72.9%) accepted the recommendation and the pregnancy was terminated, whereas 16 (27.1%) chose to continue the pregnancy (Table 1).

Renal severity, amniotic fluid status, associated abnormalities, and genetic findings are presented in Table 1. The distribution of renal severity differed between recommendation groups (*p* = 0.020). Termination was recommended in 84.2% of high-severity cases, 47.1% of moderate-severity cases, and 57.6% of low-severity cases. The rate was higher in the low- than in the moderate-severity category, largely because an additional anomaly was present in 84.8% (28/33) of low-severity but 64.7% (33/51) of moderate-severity pregnancies; among isolated renal anomalies the rates were similar (1/5 and 4/18). Amniotic fluid status was also associated with the council decision (*p* = 0.012). The recommendation rate was highest in the presence of anhydramnios (88.2%), compared with 75.0% for oligohydramnios, 51.9% for normal fluid volume, and 20.0% for polyhydramnios. In the two pregnancies with anhydramnios for which termination was not recommended, the decision reflected the advanced gestational age at diagnosis.

The recommendation also varied according to the accompanying fetal phenotype (*p* = 0.006). All fetuses with hydrops/cystic hygroma or a multiple-malformation/syndromic pattern were assigned a termination recommendation. The corresponding rates were 83.3% for skeletal or limb anomalies and 76.9% for central nervous system anomalies. By contrast, termination was recommended in 32.3% of fetuses with an isolated renal finding. Overall, the presence of an additional anomaly was associated with a higher recommendation rate than an isolated renal anomaly (68.1% vs. 32.3%, *p* = 0.002).

Genetic testing had been completed in 53 pregnancies. Termination was recommended in 77.4% of tested cases, compared with 36.0% of cases in which testing had been offered but declined (*p* < 0.001). When results were considered, 17 of 18 fetuses (94.4%) with a pathogenic or otherwise abnormal result received a termination recommendation. The rates were 66.7% among those with a normal result and 36.0% among untested pregnancies (*p* < 0.001). Testing was more frequent when an additional anomaly was present (63.9% vs. 22.6% in isolated cases), but the association persisted within strata of additional anomaly (Mantel–Haenszel odds ratio 4.57, 95% CI 1.85–11.34; *p* < 0.001), and severity was distributed similarly in tested and untested pregnancies.

The adjusted analyses are shown in Table 2. Gestational age at diagnosis remained independently associated with the council recommendation. Relative to diagnosis before 20 weeks, diagnosis at 20–23 + 6 weeks and at ≥24 weeks was associated with markedly lower odds of a termination recommendation (*p* = 0.004 and *p* < 0.001, respectively). Moderate and low renal severity were likewise associated with lower odds than high severity (*p* = 0.004 and *p* = 0.045, respectively). In contrast, an accompanying fetal anomaly increased the odds of a termination recommendation more than eightfold (adjusted odds ratio [aOR] 8.24, 95% confidence interval [CI] 1.59–42.65; *p* = 0.012), while completion of genetic testing was associated with an approximately fourfold increase (aOR 4.53, 95% CI 1.45–14.15; *p* = 0.009). The logistic model showed good explanatory performance (McFadden pseudo-R^2^ = 0.424). Estimates from the robust Poisson model were directionally consistent. Firth penalized regression gave similar estimates (additional anomaly aOR 6.01, 95% CI 1.59–32.77; genetic testing aOR 3.95, 95% CI 1.38–12.04), although confidence intervals were wide. Variance inflation factors were below 2.1. Severity and amniotic fluid status were strongly associated (Cramér’s V 0.60; 15 of 17 anhydramnios cases were high severity), and when fluid status was added the severity terms lost significance while the other estimates changed little (Appendix A, Table A2). A model including all variables with a univariable *p*-value below 0.10 (*n* = 100) gave the same pattern, and neither age nor parity was independently associated with the recommendation.

The isolated renal anomaly subgroup comprised 31 pregnancies: continuation was recommended in 21 and a termination recommendation was issued in 10 (Table 3). Within this subgroup, gestational age at diagnosis remained strongly associated with the council decision (*p* < 0.001). Renal severity was not significantly associated with the recommendation (*p* = 0.104), although the small cell counts warrant a cautious interpretation.

The distribution of individual renal diagnoses across severity categories is detailed in Appendix A (Table A1). Renal agenesis and polycystic kidney disease were the most frequent diagnoses in the high-severity group (*n* = 6 each). Multicystic dysplastic kidney (*n* = 14) and isolated pyelectasis/hydronephrosis (*n* = 13) predominated in the moderate group. Most low-severity cases consisted of isolated pyelectasis/hydronephrosis (*n* = 15) or horseshoe kidney (*n* = 8).

Postnatal outcomes were available for the 60 singleton pregnancies that continued (Table 4). Median gestational age at birth ranged from 36.0 to 38.0 weeks across severity categories and was lowest in the high-severity group. Eleven fetuses or infants (18.3%) died in utero or during the neonatal/postnatal period: one intrauterine and ten neonatal/postnatal deaths. Mortality reached 42.9% (3/7) in the high-severity group, compared with 5.3% (1/19) in the low-severity group.

The prenatal renal finding was confirmed after birth in 22 cases (36.7%) and was not confirmed in 9 (15.0%). Follow-up information was unavailable in 18 cases (30.0%). Sixteen of these pregnancies had no accessible delivery information, while two had documented birth and neonatal data but no renal-specific postnatal assessment in the council record. The prenatal finding was not confirmed after birth in 7 of 44 pregnancies (15.9%) with a continuation recommendation and in 2 of 16 (12.5%) in which the family continued despite a termination recommendation (*p* = 0.56).

## 4. Discussion

In this retrospective cohort study conducted at a tertiary perinatology council, we evaluated 103 singleton pregnancies with a prenatally diagnosed renal anomaly and found that termination was recommended in 57.3% of cases (59/103). On multivariable analysis, a later gestational age at diagnosis and a moderate or low renal severity category were independently associated with a lower likelihood of a termination recommendation, while the presence of an additional fetal anomaly (aOR 8.24) and completion of genetic testing (aOR 4.53) were independently associated with a higher likelihood. Among families advised to terminate, 72.9% accepted the recommendation and 27.1% chose to continue the pregnancy despite it. Among the 60 continued pregnancies, perinatal mortality reached 42.9% in the high-severity group, compared with 5.3% in the low-severity group.

The strong, independent relationship between gestational age at diagnosis and the termination recommendation is consistent with several large studies. Pusayapaibul et al. found that, among 461 pregnancies with a major fetal anomaly, an earlier gestational age at diagnosis was one of the strongest predictors of a termination decision [11]. A Turkish tertiary-center study likewise showed that the week of diagnosis significantly shaped the termination decision [12]. In a recent cohort of 277 CAKUT cases, Çaltek et al. also found that earlier diagnosis was independently associated with termination (adjusted OR 0.82) [4]. This pattern likely reflects both the wider window families and clinicians have to weigh their options earlier in pregnancy, and the tendency for the most lethal anomalies to be diagnosed earlier. In our own cohort, we suspect the effect of gestational age at diagnosis reflects not only the natural course of the anomaly itself but also how much decision-making time remains: for cases diagnosed before 20 weeks, additional findings that might only become apparent later in pregnancy would not yet have been observed, yet the council still had to reach a decision within a limited period, and this time pressure may itself have shaped its assessment. The earlier diagnosis in the termination-recommended group probably reflects that severe lesions, such as renal agenesis, polycystic kidney disease, and obstruction with anhydramnios, are conspicuous at the routine second-trimester scan (median gestational age at diagnosis 21.3 weeks in high-severity, 23.0 in moderate-severity, and 23.4 in low-severity cases), and that early diagnosis leaves time for genetic testing and repeated counseling.

Legal and practical constraints may also have contributed. In Turkey, termination beyond the 10th week is permitted for a high likelihood of severe disability or incurable fatal disease in the fetus, with the concurrence of a panel of specialists, and no fixed upper gestational limit has been described for such indications [12]. The time left for counseling nevertheless shrinks as pregnancy advances: a termination recommendation was issued in 95.8% of pregnancies diagnosed before 20 weeks but in 33.3% of those diagnosed from 24 weeks, which may reflect both a more favorable case mix and fewer realistic options. Our data cannot separate these influences.

The association between renal severity category, amniotic fluid status, and the termination recommendation fits with what is already known about how much prognosis depends on renal parenchymal reserve, the presence of obstruction, and amniotic fluid volume. In a recent large cohort, Arantes et al. identified oligohydramnios (HR 3.2), lower urinary tract obstruction (HR 5.1), and renal agenesis/hypodysplasia (HR 5.1) as independent risk factors for perinatal and neonatal mortality [13]. The ERKNet consensus statement similarly highlights that the development of oligohydramnios or anhydramnios is the single most important parameter for predicting fetal and neonatal death in fetuses with LUTO [14]. Claes et al. also note that, even in severe CAKUT with anhydramnios, survival is now achievable in some cases through intrauterine intervention and neonatal dialysis, though the limitations of these therapies make individualized, multidisciplinary counseling all the more important [15]. In our cohort, termination was recommended in 88.2% of anhydramnios cases, consistent with these findings; the two anhydramnios cases for whom termination was not recommended were both diagnosed late in gestation, suggesting that severity was weighed together with gestational age rather than in isolation. It is worth noting that in our council, severity grading was never based on a single sonographic measurement, but on a combined assessment of the degree of obstruction, laterality, and the amount of functioning renal tissue. Indeed, a substantial share of moderate- and low-severity cases still received a termination recommendation (47.1% and 57.6%, respectively), suggesting that severity category alone did not drive the recommendation, and that associated findings and amniotic fluid status entered the same decision alongside it. The higher rate in the low- than in the moderate-severity category reflects case mix rather than inconsistent judgment: an additional anomaly was present in 84.8% of low-severity but 64.7% of moderate-severity pregnancies, and the rates were similar within strata of anomaly status. Severity and amniotic fluid status are not independent, because fluid volume is a component of the severity grade (Cramér’s V 0.60), so we kept severity in the primary model; whether severity or fluid carries more weight in the council’s judgment cannot be resolved with this sample.

The strong relationship between the presence of an associated anomaly, completion of genetic testing, and the termination recommendation reflects the growing role genetic findings play in both prognosis and counseling in CAKUT. Koenigbauer et al. reported a pathogenic variant detection rate of 11.4% on exome sequencing in isolated CAKUT, rising to 52.6% when extrarenal findings were present [16]; this mirrors our own finding that the termination recommendation rate was markedly higher when an associated anomaly was present (68.1% vs. 32.3%). This suggests that cases referred for genetic testing likely represented more complex, or prognostically less certain, clinical pictures. Notably, in our cohort the relationship between genetic testing and the termination recommendation held regardless of the actual result: cases in which testing was offered but declined had a markedly lower termination recommendation rate (36.0%). This suggests that, in our council, genetic evaluation entered the decision not simply as a confirmatory step once a result was in hand, but as a marker of clinical complexity and prognostic uncertainty that shaped the recommendation in its own right. The association with genetic testing should not be read as a causal effect of testing. Testing was more frequent when an additional anomaly was present (86.8% of tested vs. 52.0% of untested pregnancies), so confounding by indication is likely, although the association persisted within strata of anomaly status. Declining testing may also signal a family’s wish to continue the pregnancy, and socioeconomic selection is possible for the privately funded exome sequencing; socioeconomic data were not collected.

Taken together, our findings suggest that our council did not anchor its termination recommendation to any single parameter. Rather, the recommendation appears to have been shaped by the combined clinical picture created by the timing of diagnosis, the expected renal prognosis, the broader fetal phenotype, and the availability of genetic information. The fact that all four of these variables retained independent significance in the multivariable model suggests that the council’s decision cannot be reduced to one dominant factor; instead, several overlapping but not fully redundant clinical dimensions appear to have been weighed together. In this sense, our study captures not only which factors were associated with the termination recommendation, but something of how those factors combined into the council’s overall decision-making.

The discordance we observed between the termination recommendation and the family’s actual decision (27.1%) stands out against the wider literature. In a Danish study, 92% of families offered the option of termination chose to accept it, and a lethal prognosis was the only factor associated with that decision [17]. Two recent Turkish studies speak more directly to our own findings. Başaran et al. found that families who declined termination after a fetal anomaly diagnosis most often cited religious reasons, whereas physician counseling played a larger role in the decisions of families who terminated [18]. Babadağlı et al. likewise reported that Turkish pregnant women’s preference to continue a pregnancy varied considerably by the type of anomaly involved (58.7% for anencephaly versus 92.4% for Down syndrome) and was strongly shaped by religious and cultural factors [19]. We did not record religion, socioeconomic status, or other cultural beliefs in our dataset, so we cannot directly assess what drove the 16 families who continued the pregnancy despite the council’s recommendation. In light of the studies above, however, it seems likely that a well-reasoned, clinically grounded recommendation is not, on its own, sufficient; the family’s final decision appears to pass through a set of personal and cultural considerations that lie beyond the clinical picture itself. The reasons given by families who continued despite a termination recommendation were not recorded, and the family’s decision was not modeled because only 16 families departed from the recommendation.

The prenatal–postnatal discordance and the loss to follow-up we observed among continued pregnancies are broadly consistent with the literature as well. Herthelius’s review notes that roughly a third of antenatally detected urinary tract dilatations resolve before birth, another third resolve within the first years of life, and in the remainder the dilatation either persists or a CAKUT diagnosis is confirmed [20]; this may partly explain why the prenatal finding was not confirmed postnatally in 15.0% of our cases. That 30.0% of our cases had no accessible postnatal follow-up information is a problem we suspect is common at tertiary centers generally. As Walawender et al. emphasize in their review, the long-term risk of kidney function loss across the CAKUT spectrum can only be reliably assessed through structured, sustained postnatal follow-up [21]; preventing loss to follow-up, particularly among high-severity continued pregnancies, should therefore be treated as part of the council process itself, not as an afterthought. Postnatal non-confirmation of the prenatal finding was similar whether continuation had been recommended (15.9%) or the family had continued against a termination recommendation (12.5%).

In practical terms, we suggest documenting the severity domains on the council form, offering genetic testing and counseling at the first visit so that results are available while all options remain open (with explicit counseling that a normal result does not exclude postnatal renal disease), recording the reasons behind families’ decisions, involving a psychologist or social worker, and building structured postnatal follow-up into the council process. Individualized counseling could also be supported by risk prediction models and decision aids. Models exist for specific conditions, such as postnatal renal function in lower urinary tract obstruction [22], and decision aids have been tested for prenatal testing decisions [23], but we know of no validated tool for fetal renal anomalies in general. The variables identified here could serve as candidate predictors in such a model, which would need development in a larger multicenter series and external validation.

This study has several limitations. Its single-center, retrospective design may limit how well the findings generalize elsewhere. Renal severity was categorized from the clinical assessment recorded at the council rather than from a standardized scoring system; while this arguably better reflects real clinical reasoning, it may also introduce some subjectivity. The small number of cases in the isolated renal anomaly subgroup limits the power of the severity comparison within that subgroup. The number of events was limited, producing wide confidence intervals and sparse cells despite Firth correction. Postmortem examination was not systematically performed, so antenatal diagnoses could not be verified in cases of fetal or neonatal death. Finally, we did not assess the religious, cultural, and socioeconomic factors that shape a family’s decision even after a council recommendation; as other Turkish studies have shown [18,19], this psychosocial dimension may account for a meaningful part of the decision process.

The study also has notable strengths. Every case was evaluated within a structured multidisciplinary council that included maternal–fetal medicine, pediatric nephrology, and medical genetics. Severity was graded not from the diagnostic label alone but from individual prognostic features (laterality, amniotic fluid status, and the amount of functioning renal tissue) which we believe better reflects clinical reality. Our study also looked beyond the council’s recommendation to examine the family’s actual concordance with it, allowing us to quantify the gap between the clinical decision and the decision families ultimately made. Finally, using conventional logistic, robust-variance Poisson, and Firth penalized logistic regression showed that our findings held up across three different estimation approaches.

In conclusion, the termination recommendation issued by our council for fetuses with a renal anomaly was shaped by the timing of diagnosis, the expected renal prognosis, the broader fetal phenotype, and the availability of genetic information. Yet the clear gap between the council’s recommendation and the family’s ultimate decision suggests that psychosocial and cultural factors, beyond the purely clinical ones, also play a role in this process. The variable postnatal course among continued pregnancies further supports the value of individualized counseling within a structured multidisciplinary follow-up framework. The wide confidence intervals call for cautious interpretation, and validated prediction and decision-support tools are needed.

## Figures and Tables

**Figure 1:**
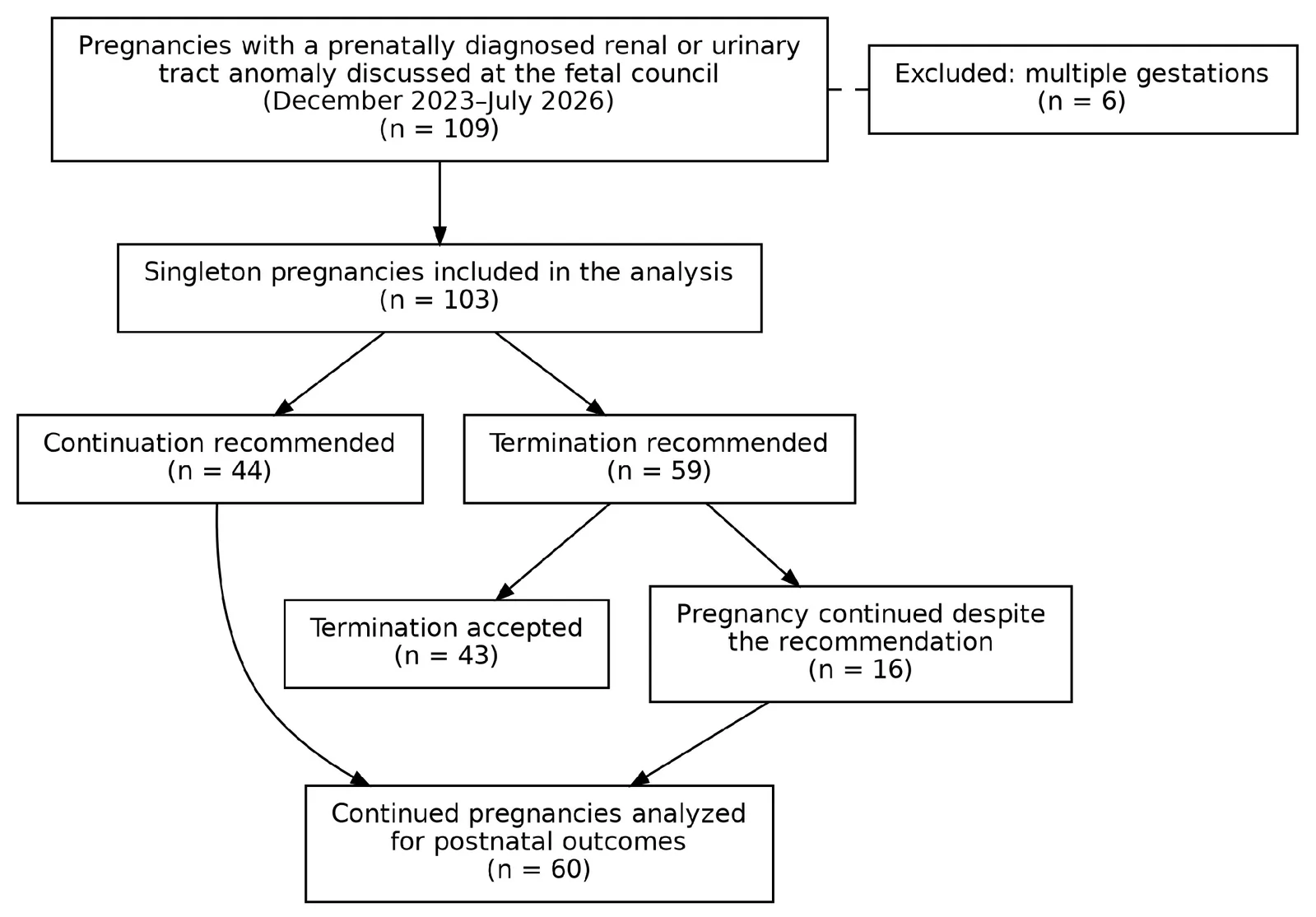
Flow diagram of pregnancies with a prenatally diagnosed renal or urinary tract anomaly discussed at the fetal council, including the council recommendation and the family’s subsequent decision.

**Table 1:** Characteristics of the pregnancies according to the fetal council recommendation.

Variable	Continuation Recommended (*n* = 44)	Termination Recommended (*n* = 59)	Overall *n* (Recommendation Rate)	*p*-Value
Maternal age, years, median (IQR)	30 (27–33)	27 (23–31)		0.060
Gravidity, median (IQR)	2 (2–3)	2 (1–3)		0.017
Parity, median (IQR)	1 (1–2)	0 (0–1)		0.003
Gestational age at diagnosis, weeks, median (IQR)	25 (23–31)	21 (18–24)		<0.001
Gestational-age category, *n* (%)				<0.001
<20 weeks	1 (2.3)	23 (39.0)	24 (95.8)	
20–23 + 6 weeks	15 (34.1)	22 (37.3)	37 (59.5)	
≥24 weeks	28 (63.6)	14 (23.7)	42 (33.3)	
Renal severity, *n* (%)				0.020
High	3 (6.8)	16 (27.1)	19 (84.2)	
Moderate	27 (61.4)	24 (40.7)	51 (47.1)	
Low	14 (31.8)	19 (32.2)	33 (57.6)	
Amniotic fluid status, *n* (%)				0.012
Anhydramnios	2 (4.5)	15 (25.4)	17 (88.2)	
Normal	37 (84.1)	40 (67.8)	77 (51.9)	
Oligohydramnios	1 (2.3)	3 (5.1)	4 (75.0)	
Polyhydramnios	4 (9.1)	1 (1.7)	5 (20.0)	
Associated finding: dominant category, *n* (%)				0.006
Gastrointestinal anomaly	4 (9.1)	5 (8.5)	9 (55.6)	
Hydrops fetalis/cystic hygroma	0 (0.0)	4 (6.8)	4 (100.0)	
Cardiac anomaly	4 (9.1)	8 (13.6)	12 (66.7)	
Multiple malformations/syndromic pattern	0 (0.0)	6 (10.2)	6 (100.0)	
Central nervous system anomaly	3 (6.8)	10 (16.9)	13 (76.9)	
Soft marker only	11 (25.0)	11 (18.6)	22 (50.0)	
None	21 (47.7)	10 (16.9)	31 (32.3)	
Skeletal/limb anomaly	1 (2.3)	5 (8.5)	6 (83.3)	
Any additional anomaly, *n* (%)				0.002
Present	23 (52.3)	49 (83.1)	72 (68.1)	
Absent	21 (47.7)	10 (16.9)	31 (32.3)	
Genetic testing, *n* (%)				<0.001
Performed	12 (27.3)	41 (69.5)	53 (77.4)	
Offered but declined	32 (72.7)	18 (30.5)	50 (36.0)	
Genetic test result, *n* (%)				<0.001
Normal	11 (25.0)	22 (37.3)	33 (66.7)	
Pathogenic/abnormal	1 (2.3)	17 (28.8)	18 (94.4)	
Result unavailable/unknown	0 (0.0)	2 (3.4)	2 (100.0)	
Not tested	32 (72.7)	18 (30.5)	50 (36.0)	
Parental response to the council recommendation, *n* (%)				
Pregnancy continued	44 (100.0)	16 (27.1)		
Pregnancy terminated	0 (0.0)	43 (72.9)		
Unknown	0 (0.0)	0 (0.0)		

Values are *n* (%). Continuous variables are median (IQR) and were compared with the Mann–Whitney U test; categorical variables were compared with the chi-square test. In the overall column, percentages are the proportion receiving a termination recommendation within each category (row percentage); in the two recommendation columns, percentages describe the distribution within each group (column percentage).

**Table 2:** Multivariable analysis of factors associated with a recommendation for termination.

Variable	Logistic aOR (95% CI)	*p*-Value	Firth aOR (95% CI)	*p*-Value	Poisson aRR (95% CI)	*p*-Value
Intercept	50.08 (3.09–812.09)	0.006	24.60 (3.01–400.58)	0.001	0.65 (0.41–1.02)	0.062
Diagnosis at 20–23 + 6 weeks (ref: <20 weeks)	0.02 (0.00–0.28)	0.004	0.04 (0.00–0.28)	<0.001	0.64 (0.49–0.83)	<0.001
Diagnosis at ≥24 weeks (ref: <20 weeks)	0.01 (0.00–0.13)	<0.001	0.02 (0.00–0.14)	<0.001	0.44 (0.29–0.66)	<0.001
Moderate severity (ref: high)	0.06 (0.01–0.41)	0.004	0.09 (0.01–0.43)	0.002	0.54 (0.40–0.75)	<0.001
Low severity (ref: high)	0.13 (0.02–0.96)	0.045	0.18 (0.02–0.96)	0.045	0.68 (0.49–0.95)	0.024
Additional anomaly present (ref: absent)	8.24 (1.59–42.65)	0.012	6.01 (1.59–32.77)	0.007	1.70 (1.07–2.70)	0.023
Genetic testing performed (ref: not performed/declined)	4.53 (1.45–14.15)	0.009	3.95 (1.38–12.04)	0.010	1.64 (1.14–2.35)	0.008

Logistic regression: *n* = 103; McFadden pseudo-R^2^ = 0.424. Firth, penalized-likelihood logistic regression with profile-likelihood confidence intervals. Relative risks were estimated using Poisson regression with robust (sandwich) variance. CI, confidence interval; OR, odds ratio; RR, relative risk.

**Table 3:** Factors associated with the fetal council recommendation in the isolated renal anomaly subgroup (*n* = 31).

Variable	Continuation Recommended (*n* = 21)	Termination Recommended (*n* = 10)	*p*-Value
Gestational-age category, *n* (%)			<0.001
<20 weeks	1 (4.8)	6 (60.0)	
20–23 + 6 weeks	5 (23.8)	3 (30.0)	
≥24 weeks	15 (71.4)	1 (10.0)	
Renal severity, *n* (%)			0.104
High	3 (14.3)	5 (50.0)	
Moderate	14 (66.7)	4 (40.0)	
Low	4 (19.0)	1 (10.0)	

Gestational age at diagnosis also differed when analyzed as a continuous variable (Mann–Whitney U test, *p* < 0.001). The severity comparison should be interpreted cautiously because of the small cell counts.

**Table 4:** Postnatal outcomes among continued pregnancies according to prenatal renal severity (*n* = 60).

Prenatal Severity	*n*	GA at Birth, Weeks	Apgar 1 min	Apgar 5 min	Birth Weight, g	NICU Admission	Intrauterine/Postnatal Death	Postnatally Confirmed	Postnatal Normal	Follow-Up Unavailable
High	7	36.0 (26.0–37.1)	6 (3–8)	7 (3–9)	2100 (950–3600)	4/4	3 (42.9%)	3 (42.9%)	0 (0.0%)	1 (14.3%)
Moderate	34	38 (27–39)	7 (2–8)	8.5 (5–9)	3100 (970–5420)	11/12	7 (20.6%)	15 (44.1%)	4 (11.8%)	8 (23.5%)
Low	19	37.5 (26.0–39.9)	8 (1–8)	9 (1–9)	2900 (670–3700)	5/6	1 (5.3%)	4 (21.1%)	5 (26.3%)	9 (47.4%)
Total	60	—	—	—	—	—	11 (18.3%)	22 (36.7%)	9 (15.0%)	18 (30.0%)

Continuous variables are presented as median (minimum–maximum); categorical variables are shown as *n* (%) or *n*/*N* when data were incomplete. NICU admission is reported using available-case denominators. “Postnatally confirmed” indicates verification of the prenatal renal finding on postnatal imaging or follow-up. The unavailable-follow-up group included 16 cases without delivery information and 2 cases with documented birth data but no renal-specific postnatal result.

## Data Availability

The data presented in this study are available on request from the corresponding author, subject to institutional and ethical restrictions on sharing identifiable clinical data.

## Abbreviations

The following abbreviations are used in this manuscript:

aOR	adjusted Odds Ratio
CAKUT	Congenital Anomalies of the Kidney and Urinary Tract
CI	Confidence Interval
GA	Gestational Age
HR	Hazard Ratio
LUTO	Lower Urinary Tract Obstruction
MCDK	Multicystic Dysplastic Kidney
NICU	Neonatal Intensive Care Unit
OR	Odds Ratio
PUV	Posterior Urethral Valves
RR	Relative Risk
UPJ	Ureteropelvic Junction

## Appendix A

Table A1 describes the severity categories used in this study, and Table A2 reports the sensitivity analyses of the multivariable models.

**Table A1:** Domains and definitions of the prenatal renal severity categories, with the characteristics and renal diagnoses observed in each category.

Characteristic	High Severity (*n* = 19)	Moderate Severity (*n* = 51)	Low Severity (*n* = 33)
Council definition	Risk of profound renal dysfunction, pulmonary hypoplasia, or death	Meaningful likelihood of postnatal morbidity, surveillance, or intervention	Expected favorable course with limited functional impairment
Bilateral involvement, *n* (%)	18 (94.7)	27 (52.9)	26 (78.8)
Anhydramnios or oligohydramnios, *n* (%)	17 (89.5)	3 (5.9)	1 (3.0)
Gestational age at diagnosis, weeks, median	21.3	23.0	23.4
Renal diagnosis, *n*			
LUTO (PUV/megacystis)	2	6	0
Horseshoe kidney	0	0	8
Other renal abnormality	2	2	7
Ectopic kidney	0	3	0
MCDK	2	14	0
Polycystic kidney disease	6	4	0
Renal agenesis	6	6	1
Renal mass	0	1	0
UPJ obstruction	0	2	2
Isolated pyelectasis/hydronephrosis	1	13	15
Additional anomaly present, *n* (%)	11 (57.9)	33 (64.7)	28 (84.8)
Termination recommended, *n* (%)	16 (84.2)	24 (47.1)	19 (57.6)

Severity was assigned by council consensus, without numerical cut-offs, after weighing laterality, functioning renal tissue, obstruction, amniotic fluid volume, expected renal function, need for neonatal treatment, and anticipated survival. The definitions in the second row are the council’s; the other rows describe the cases as graded and are not grading rules. Bilateral includes fusion anomalies; laterality was unspecified in one high- and three moderate-severity cases. LUTO, lower urinary tract obstruction; MCDK, multicystic dysplastic kidney; PUV, posterior urethral valves; UPJ, ureteropelvic junction.

**Table A2:** Sensitivity analyses of the multivariable models (Firth penalized logistic regression).

Variable	Primary Model Plus Fluid Status: aOR (95% CI)	*p*-Value	All Variables with *p* < 0.10: aOR (95% CI)	*p*-Value
Diagnosis at 20–23 + 6 weeks (ref: <20 weeks)	0.03 (0.00–0.22)	<0.001	0.04 (0.00–0.36)	0.002
Diagnosis at ≥24 weeks (ref: <20 weeks)	0.01 (0.00–0.11)	<0.001	0.03 (0.00–0.22)	<0.001
Moderate severity (ref: high)	0.36 (0.03–3.50)	0.374	0.39 (0.03–4.54)	0.454
Low severity (ref: high)	0.80 (0.07–7.96)	0.847	0.82 (0.06–9.87)	0.880
Anhydramnios or oligohydramnios (ref: normal or polyhydramnios)	7.82 (0.95–113.90)	0.056	6.82 (0.75–107.47)	0.090
Additional anomaly present (ref: absent)	8.88 (1.99–81.79)	0.003	8.93 (1.97–82.69)	0.003
Genetic testing performed (ref: not performed/declined)	3.58 (1.24–11.04)	0.019	3.93 (1.26–13.45)	0.018
Maternal age, per year	—	—	0.95 (0.85–1.07)	0.432
Parity, per birth	—	—	0.85 (0.42–1.65)	0.625

Firth penalized logistic regression with profile-likelihood confidence intervals. The first model (*n* = 103) adds anhydramnios or oligohydramnios to the primary model; the second (*n* = 100; three pregnancies with missing parity excluded) includes all variables with a univariable *p*-value below 0.10.
